# The Impact of Weekday-to-Weekend Sleep Differences on Health Outcomes among Adolescent Students

**DOI:** 10.3390/children9010052

**Published:** 2022-01-03

**Authors:** Jinseok Kim, Jin-Won Noh, Ahraemi Kim, Young Dae Kwon

**Affiliations:** 1 Department of Social Welfare, Seoul Women’s University, Seoul 01797, Korea; jskim@swu.ac.kr (J.K.); akim@swu.ac.kr (A.K.); 2 Division of Health Administration, College of Software and Digital Healthcare Convergence, Yonsei University, Wonju 26493, Korea; jinwon.noh@gmail.com; 3 Department of Humanities and Social Medicine, College of Medicine and Catholic Institute for Healthcare Management, The Catholic University of Korea, Seoul 06591, Korea

**Keywords:** adolescent students, weekday-to-weekend sleep differences, health outcomes

## Abstract

The sleep difference between weekdays and weekends can lead to negative physical and mental health outcomes in adolescents. Thus, this study has attempted to analyze the impact of sleep time differences on various health outcomes, using nationally representative panel data. Data from the junior high school student panel of the Korean Children and Youth Panel Survey were analyzed. The sleep difference was defined as the difference between the average sleep duration on weekdays and that on weekends in minutes. A series of mixed effect linear regression models for continuous variables or mixed effect logit regression for binary variables was utilized. Korean adolescent students reported from 96.8 min to 133.2 min of sleep duration difference between weekdays and weekends. After controlling for gender, parent work status, and type of housing, the weekday-to-weekend sleep differences were associated with various health-related outcomes including concentration difficulty, aggression, somatic symptoms, and withdrawal. Additionally, adolescent student life satisfaction was associated with sleep difference. The sleep differences among adolescent students were more associated with mental health-related outcomes and emotional symptoms than with physical health-related outcomes. The appropriate intervention to reduce the sleep difference gap is an important key to improve health in the adolescence period.

## 1. Introduction

Adolescent sleep quality is crucial in their daily lives as it substantially affects healthy development. Unfortunately, however, adolescents have chronically experienced a low quality of sleep patterns in the current social environments. Growing research notes that social jetlag is one of the negative sleep patterns that affect adolescent health [1,2]. Sleep debt is the discrepancy in individual preference for sleep timing or chronotype and actual sleep duration. However, social jetlag represents the difference between biological timing and social rhythms [3,4]. Social jetlag of adolescents has been measured as the discrepancy in sleep timing between weekdays and weekends [5,6]. 

Further, the discrepancy during school terms becomes more common [1,5]. For instance, 35–40% of US secondary school students experience over two hours of weekend oversleep [2], while the average hours of weekend oversleep among Korean adolescents is slightly over two hours [7]. As sleep time difference between weekdays and weekends is problematic and conspicuous [8,9], weekday-to-weekend sleep differences among adolescents are receiving increasing attention from researchers.

Weekday-to-weekend sleep differences in adolescent students are linked to early rise time on weekdays, late bedtime on weekdays, and weekend oversleep. The difference in sleep timing is affected by factors including heavy study burden and many extracurricular activities, and the difference is more evident during school terms. In the highly competitive education environments, 74.3% of Korean adolescents participate in extracurricular activities and spend 6.5 weekly hours on them [10]. This has been widely known as the main factor of sleep difference among adolescents in Korea [7,11]. Additionally, adolescents are inclined to have circadian delay (e.g., an increase in eveningness) due to the developmental characteristics of their age groups [5]. Increased use of digital devices (e.g., smartphone, tablet PC, and computer) has widened the difference in sleep timing [1].

Weekday-to-weekend sleep difference has led to negative outcomes pertaining to physical and mental health. Weekday-to-weekend sleep difference is more likely to cause diverse mental health outcomes including depression [7,11,12], aggression [10], concentration difficulty [13], social withdrawal [14,15], and somatic symptoms [11,16]. However, the literature is limited in that many studies focus more on the relationship between difference in sleep timing and depression than the relationships with other mental health outcomes. Difference in sleep timing relates to physical health outcomes including chronic symptoms and obesity [7,17]. Obesity has been addressed in the literature in terms of body mass index (BMI) [18], obesity [6,7], and overweight [19]. Previous studies examined the effects of adolescents’ weekday-to-weekend sleep difference on self-rated health [20] and life satisfaction [21].

Weekday-to-weekend sleep differences of adolescent students and its effects rely on social and cultural factors as extracurricular activities, school schedules, and accessibility of digital devices. Prior research shows that Black adolescents slept less than White adolescents [2], and Korean adolescents have severe sleep deprivation on school days [13]. Sun et al. (2019) indicated that Asian adolescences’ social jetlag was positively associated with depressive symptoms and obesity [5]. However, the effects of social jet lag on health outcomes among Korean adolescents remain unclear. It is crucial to understand the relationships in Korean academic elitism culture and compare the findings to the prior literature. Thus, the findings can give fruitful discussions to grasp how sleep difference affects health outcomes in diverse cultural contexts. In light of these contexts, this study aims to examine the impact of the sleep time difference between weekday and weekend on various health outcomes using nationally representative panel data from Korean adolescent students.

## 2. Materials and Methods

### 2.1. Data and Participants

Data from the Korean Children and Youth Panel Survey (KCYPS), a nationally representative study of Korean children and youths, were utilized for this study. The KCYPS was designed to investigate multiple aspects of adolescent growth and development. It started to collect data in 2010, from three panels of first and fourth grade elementary school students, as well as first grade junior high school students (*n* = 7071), which was followed annually until 2016. The KCYPS implemented a multi-stage stratified cluster sampling method with schools as the primary sampling unit. Among the three panels, that of junior high school students (*n* = 2351) was used in this analysis and included participant developmental stages from first year of junior high school up to one year after high school graduation. 

### 2.2. Variables and Measurements

The sleep duration was calculated using the answers to the question “What time do you usually go to bed and get up?”, which was asked for weekdays and for weekends separately. The sleep difference between weekdays and weekends was defined as the difference between the average sleep duration on weekdays and that on weekends in minutes. Specifically, the sleep difference was calculated by subtracting the average weekdays sleep duration from the average weekends sleep duration in minutes. 

Several psychological outcomes such as concentration difficulty, aggression, somatic symptoms, withdrawal, depression, and health-related outcomes such as self-rated health, number of chronic symptoms, and obesity were included in this analysis. The measures of concentration difficulty, aggression, and somatic symptoms were comprised of seven items, six items, and eight items, respectively, and adopted from the Emotional or Behavioral Problems Scale [22] measured in a four-point Likert scale. Youth withdrawal was measured using a subset of the Behavior Problem Scale for Children and Adolescence [23] comprised of five items measured in a four-point Likert scale. Depression of the youths was measured using the Symptom Checklist [24] comprised of 10 items also measured in a four-point Likert scale. Self-rated health was measured using a four-point Likert scale (1 = ‘very unhealthy’; 4 = ‘very healthy’). Number of chronic symptoms was measured by counting the number of chronic symptoms the respondents had among asthma, rhinitis, atopic dermatitis, heart disease, diabetes, or others during the previous year, which were answered by the respondents. BMI was calculated using self-reported height and weight and was used to define obesity status [25]. Adolescent life satisfaction was measured in Waves 1 and 3–6, while BMI was measured in Waves 2–6. The number of chronic symptoms were measured only in Waves 1 and 4.

Gender, type of housing, and parents’ work statuses were included as control variables. Gender (male = 1, female = 0) and parents’ work statuses (working = 1, not working = 0) were dichotomized. Finally, type of housing had three categories (1 = house, 2 = apartment, 3 = others).

### 2.3. Statistical Analysis

A set of descriptive analyses was conducted to provide overall characteristics of the sample. The hypothesized relationships of sleep patterns with health outcomes were tested using mixed effect linear regression models for continuous variables or mixed effect logit regression for binary variables. The multi-level regression models can be written as follows:

$${\left[health\;outcome\right]}_{ij}=\beta_{0j}^{1}+\beta_{1j}^{1}{\left[sleep\;difference\right]}_{ij}+\sum_{k=2}^{l}\beta_{kj}^{1}{\left[covariates\right]}_{ij}^{k}+\varepsilon_{ij}^{1}\;\mathrm{if}\;[health\;outcome]\mathrm{is}\mathrm{continuous}$$

$$ogit{\left[health\;outcome\right]}_{ij}=\beta_{0j}^{1}+\beta_{1j}^{1}{\left[sleep\;difference\right]}_{ij}+\sum_{k=2}^{l}\beta_{kj}^{1}{\left[covariates\right]}_{ij}^{k}\;\mathrm{if}\;[health\;outcome]\mathrm{is}\mathrm{discrete}$$

where $\beta_{0j}^{1}=\gamma_{00}^{1}+u_{0j}^{1};\;u_{0j}^{1}\sim N\left(0,\tau_{00}^{1}\right);\;\beta_{lj}^{m}=\gamma_{l0}^{m}\;if\;l\neq 0,\;$ and $\varepsilon_{0j}^{1}\sim N\left(0,{\left(\sigma^{1}\right)}^{2}\right)$.

In the equation above, $\gamma ’s_{}^{}$ are for fixed effects and u’s and $\varepsilon ’s_{}^{}$ for random effects in the second level and in the first level, respectively. The mixed effect regression models were considered appropriate for analysis of panel data structure, such as the KCYPS [26]. Stata Statistical Software Release 16 (StataCorp, College Station, TX, USA) was used to manipulate the data and calculate the model parameters. 

## 3. Results

The characteristics of the sample are presented in Table 1. Males and females were almost evenly distributed throughout the study period. About two-thirds of mothers were working outside the home (minimum: 62.5% [Wave 1], maximum: 70.9% [Wave 5]), while over 90% of fathers were working outside the home (minimum: 90.4% [Wave 6], maximum: 98.4% [Wave 2]). About 60% of adolescent students responded that both their parents were working (minimum: 58.8% [Wave 6], maximum: 68.1% [Wave 5]). Most adolescent students were living in an apartment (minimum: 59.1% [Wave 1], maximum: 60.7% [Wave 3]), and about 20% were living in housing types other than a house or apartment (minimum: 14.6% [Wave 5], maximum: 20.3% [Wave 1]). 

Throughout the study period, the adolescent students reported from 96.8 min (SD = 102.4) at Wave 1 to 133.2 min (SD = 103.4) at Wave 4 of sleep duration difference between weekdays and weekends. Table 1 summarizes the measures of emotional status including concentration difficulty, aggression, somatic symptoms, withdrawal, and depression, which were measured on four-point Likert scales in Waves 2–4 and 6. 

Table 2 summarizes the results of the mixed effects regression analyses of health-related outcomes with weekday-to-weekend sleep difference as a predictor after controlling for gender, parents’ work status, and type of housing. Results indicate that adolescent students who reported larger weekday-to-weekend sleep duration difference presented greater difficulty in concentration (B (SE) = 0.012 (0.003), *p* < 0.01), aggression (B (SE) = 0.011 (0.003), *p* = 0.01), somatic symptoms (B (SE) = 0.014 (0.003), *p* < 0.01), and withdrawal (B (SE) = 0.008 (0.004), *p* = 0.036). Adolescent students’ weekday-to-weekend sleep difference showed a significant association with overall emotional symptoms (B (SE) = 0.010 (0.002), *p* < 0.01) but not with depression (B (SE) = 0.004 (0.003), *p* = 0.161). Additionally, adolescent student life satisfaction was negatively associated with sleep difference (B (SE) = −0.009 (0.003), *p* < 0.01), but SRH status was not (B (SE) = 0.002 (0.003), *p* = 0.527). Neither a larger number of chronic symptoms experienced (B (SE) = 0.010 (0.006), *p* = 0.081) nor BMI (B (SE) = −0.065 (0.051), *p* = 0.202) was associated with weekday-to-weekend sleep duration difference among adolescent students (Table 2).

## 4. Discussion

This study analyzed the impact of the sleep time difference on various health outcomes among adolescent students. Higher sleep difference of weekday-to-weekend was associated with increased concentration difficulty, aggression, somatic symptoms, withdrawal, life satisfaction, and all emotional outcomes. This study showed that concentration difficulty had a significant relationship with sleep difference. Previous studies have reported a consistent relationship between academic concentration and sleep in adolescent students. In particular, weekend sleep delay (i.e., weekday-to-weekend difference in sleep timing) had a stronger adverse effect on academic performance, substance use, and body weight compared with weekend catch-up sleep (i.e., weekday-to-weekend difference in sleep duration) [5]. This negative effect could be explained by the reduced cognitive abilities due to weekday-to-weekend sleep differences. When adolescents had recovery sleep on weekends, it has been shown that deteriorated neurobehavioral functions were incompletely recovered [5,27]. Agostini et al. (2017) reported that adolescents were exposed to delayed bedtime and reduced total sleep time due to biological and social factors [27]. These deficits of sleep can lead to decreased memory consolidation, negative emotions, and worse performance. Declined sleep onset latency and increased total sleep time has been found on sleep recovery nights, resulting in weekday-weekend sleep differences [28,29].

Students who had higher weekday-to-weekend sleep differences were more likely to experience social withdrawal. Previous studies indicated that social withdrawal could be induced by daytime sleepiness, which was caused by later onset of sleep on weekday and accumulation of sleep debt, in response to academic and social demands [30]. In the United States, school start times are a huge contributor to sleep debt which is induced due to difference in individual preferred sleep timing and actual sleep duration, particularly among high school students [4,31,32]. The mechanisms underlying the association between sleep and social withdrawal were impairment of prosocial signal parts in the brain that made it difficult to understand the intentions of others [15]. Another study reported that individuals with a secure social environment had less sleep fragmentation than lonely individuals [33]. 

This suggests that perceptions of a secure social environment promote better sleep.

The discrepancy of weekday and weekend sleep was associated with a higher degree of aggression and all emotional outcomes in this study. Sleep duration was the best statistical predictor for verbal aggression and anger. In addition, social jetlag which is likely to have drastic variability due to discrepancy of biological and social rhythms was related to physical aggression, and morningness-eveningness was related to hostility [10,34]. Those with evening preferences tend to have larger discrepancies in the weekday and weekend sleep durations, particularly when school start times are earlier. It is possible that the circadian preference is the mechanism through which the relations found are explained. Those who perceived higher social jetlag and could not navigate environmental demands can have increased stress and anxiety [10,35]. Those findings indicate that shorter total sleep time on school nights and greater social jetlag increases the risk of anxiety symptoms in adolescents [10,35]. These results can be explained by the vulnerability of adolescent emotional reactivity [36,37]. Adolescents can experience an increased change of emotion and might not be able to regulate both behavior and emotions compared to other periods [36,37]. In addition, according to Sleep in America Poll research, 55% of individuals responded that sleepiness was related to their moods, such as irritability [38].

In this study, somatic symptoms were associated with sleep difference. These results support the prior research indicating that a longer tutoring time outside school was related with increasing somatic symptoms because of its effect of a decrease in sleep duration [39]. Moreover, it has been explained that chronic work stress was one of the factors increasing somatic complaints [40]. However, in this study, the physical health outcomes including chronic symptoms and BMI status were not associated with sleep difference. Physical health showed no statistical significance with Sleep Health Composite Score, which included duration, timing, regularity, and efficiency of sleep [41]. Prior studies showed inconsistent results for this relationship. Asian adolescents who had sleep difference were less likely to be overweight and obese [5]. However, this result could be explained by sleep variability as an inconclusive factor for metabolic function in adolescents [42,43].

Lower life satisfaction was associated with higher weekday and weekend sleep difference. Evening persons have a later endogenous circadian rhythm, making it more difficult to entrain to the external 24-h light dark cycle. This makes these people often sleep more on the weekends. This was also consistent with previous research that suggested those having eveningness, which is induced by inconsistency of personal and environmental rhythms, were more likely to have lower life satisfaction [21,44]. The misalignment of endogenous rhythm and environmental demands leads to larger weekday and weekend sleep differences and the relationship between morningness and higher life satisfaction was similar across countries [5,44,45].

There were several limitations in this study. First, this study used data from self-reported questionnaire, so the findings of sleep difference could have recall bias. Therefore, future studies should consider objective methods to measure sleep time. Further, this study used data from only adolescents who attend school. Therefore, it is difficult to generalize the results of this study, and it might have bias. In addition, adolescents’ circadian-driven preference for sleep timing was not included, and adolescents who were chronically sleep deprived were not considered. The further studies need to consider the type of sleep difference. Lastly, the relationship between sleep and somatic symptoms could show reverse causality. Despite these limitations, this study provides meaningful results on health outcomes associated with sleep difference between weekday and weekend among adolescent students, using a longitudinal design that could be interpreted as causality. In addition, this longitudinal study suggests the importance of understanding weekday-to-weekend sleep difference on health-related outcomes in adolescent students.

## 5. Conclusions

In the nationally representative panel data from Korean adolescent students, weekday-to-weekend sleep differences are related to various health outcomes. This sleep difference was associated with increased concentration difficulty, aggression, somatic symptoms, withdrawal, all emotional outcomes, and decreased life satisfaction. Adolescents are more likely to have greater sleep differences between weekdays and weekend and be more emotionally reactive than other age groups. These findings provide important information for improving adolescent overall health and healthy sleep patterns. Indeed, adolescents are more likely to have sleep variability and vulnerability of emotional reactivity. Therefore, policy makers should focus on adolescent sleep differences and recognize the importance of sleep patterns in various health outcomes among adolescents. 

There is a need for education that emphasizes the importance of proper sleep pattern on weekday-weekend for adolescents. Short- and long-term policies that manage school time and weekday extra-school tutoring time for reducing the difference in sleep time among adolescent students are necessary to improve health outcomes and academic performance.

## Figures and Tables

**Table 1:** Sample characteristics of Korean adolescent students.

	Wave 1		Wave 2		Wave 3		Wave 4		Wave 5		Wave 6	
	*n*	%	*n*	%	*n*	%	*n*	%	*n*	%	*n*	%
Gender: female	1175	50.0	1128	49.5	1119	49.5	1033	49.0	1024	49.0	1015	49.4
Mother working	1376	62.5	1401	67.3	1422	68.3	1350	68.3	1346	70.9	1269	65.1
Father working	2101	96.5	2018	98.4	1999	97.4	1909	97.4	1820	97.5	1761	90.4
Both parents working	1211	59.1	1266	64.9	1262	65.0	1220	65.8	1203	68.1	1140	58.8
Housing type: apartment	1385	59.1	1327	59.8	1349	60.7	1249	59.2	1226	60.4	1188	60.6
Housing type: other	477	20.3	362	16.3	337	15.2	357	16.9	296	14.6	327	16.7
	**Mean**	**SD**	**Mean**	**SD**	**Mean**	**SD**	**Mean**	**SD**	**Mean**	**SD**	**Mean**	**SD**
Sleep differences (minute)	96.8	102.4	103.7	101.1	106.2	96.9	133.2	103.4	132.6	99.5	116.2	100.2
Concentration difficulty			2.4	0.5	2.5	0.6	2.2	0.5			2.1	0.5
Aggression			2.1	0.6	2.2	0.6	2.0	0.5			1.9	0.5
Somatic symptoms			2.0	0.6	2.1	0.6	2.0	0.5			1.9	0.6
Withdrawal			2.2	0.7	2.3	0.7	2.2	0.7			2.3	0.7
Depression			1.9	0.6	2.0	0.6	1.9	0.6			1.9	0.5
Emotional outcomes: all			2.1	0.5	2.2	0.5	2.0	0.4			2.0	0.4
Life satisfaction	2.8	0.7			2.9	0.7	2.8	0.6	2.8	0.6	2.9	0.6
Self-rated health	3.2	0.6	3.2	0.6	3.2	0.6	3.2	0.6	3.1	0.6	3.2	0.6
Number of chronic symptoms	0.6	0.8					0.6	0.8				
Body mass index			−2.0	15.3	−1.1	14.8	1.8	15.3	3.2	15.3	5.7	16.2

SD, standard deviation; The participants were in their first year of junior high school at Wave 1 and third year of high school at Wave 6.

**Table 2:** Mixed effects regression analyses of health-related outcomes and emotional symptoms with weekday-to-weekend sleep duration difference.

Outcome Variables	B	SE (B)
Concentration difficulty	0.012 ***	(0.003)
Aggression	0.011 ***	(0.003)
Somatic symptoms	0.014 ***	(0.003)
Withdrawal	0.008 **	(0.004)
Depression	0.004	(0.003)
Emotional outcomes: all	0.010 ***	(0.002)
Life satisfaction	−0.009 ***	(0.003)
Self-rated health	0.002	(0.003)
Chronic symptoms	0.010 *	(0.006)
Body mass index	−0.065	(0.051)

* *p* < 0.10, ** *p* < 0.05, *** *p* < 0.01. Note: results were controlled for gender, parent’s work status, and type of housing (i.e., house/apartment/and others). SE, standard error.

## Data Availability

Publicly available datasets were analyzed in this study. This data can be found here: https://www.nypi.re.kr/archive/eps (accessed on 15 November 2021).

## References

[1] Henderson S.E., Brady E.M., Robertson N. (2019). Associations between social jetlag and mental health in young people: A systematic review. Chronobiol. Int..

[2] Matthews K.A., Hall M., Dahl R.E. (2014). Sleep in healthy black and white adolescents. Pediatrics.

[3] Roenneberg T., Wirz-Justice A., Merrow M. (2003). Life between clocks: Daily temporal patterns of human chronotypes. J. Biol. Rhythm..

[4] Silva C.M., Mota M.C., Miranda M.T., Paim S.L., Waterhouse J., Crispim C.A. (2016). Chronotype, social jetlag and sleep debt are associated with dietary intake among Brazilian undergraduate students. Chronobiol. Int..

[5] Sun W., Ling J., Zhu X., Lee T.M.-C., Li S.X. (2019). Associations of weekday-to-weekend sleep differences with academic performance and health-related outcomes in school-age children and youths. Sleep Med. Rev..

[6] Roenneberg T., Allebrandt K.V., Merrow M., Vetter C. (2012). Social jetlag and obesity. Curr. Biol..

[7] Ryu H.-R., Kim I.-Y., Suh S. (2015). Gender differences in the relationship between social jet lag, depression, and obesity in Korean children and adolescents. J. Sleep Med..

[8] Randler C., Vollmer C. (2013). Aggression in young adults—A matter of short sleep and social jetlag?. Psychol. Rep..

[9] Levandovski R., Dantas G., Fernandes L.C., Caumo W., Torres I., Roenneberg T., Hidalgo M.P.L., Allebrandt K.V. (2011). Depression scores associate with chronotype and social jetlag in a rural population. Chronobiol. Int..

[10] Ministry of Education 2019 Private Education Expenditure. http://kostat.go.kr/portal/eng/pressReleases/11/2/index.board.

[11] Kim K.H. (2017). Factors influencing high school students’ sleep duration: Analyzing the 5th wave data from Korean Children & Youth Panel Study. Korean J. Youth Welf..

[12] Mathew G.M., Hale L., Chang A.-M. (2019). Sex moderates relationships among school night sleep duration, social jetlag, and depressive symptoms in adolescents. J. Biol. Rhythm..

[13] Kim S.J., Lee Y.J., Cho S.-J., Cho I.-H., Lim W., Lim W. (2011). Relationship between weekend catch-up sleep and poor performance on attention tasks in Korean adolescents. Arch. Pediatr. Adolesc. Med..

[14] Rondon A.T., Hilton D.C., Jarrett M.A., Ollendick T.H. (2020). Sleep, internalizing problems, and social withdrawal: Unique associations in clinic-referred youth with elevated sluggish cognitive tempo symptoms. J. Atten. Disord..

[15] Simon E.B., Walker M.P. (2018). Sleep loss causes social withdrawal and loneliness. Nat. Commun..

[16] Maultsby K.D., Luk J.W., Sita K.R., Lewin D., Simons-Morton B.G., Haynie D.L. (2021). Three dimensions of sleep, somatic symptoms, and marijuana use in US High school students. J. Adolesc. Health.

[17] Stoner L., Castro N., Signal L., Skidmore P., Faulkner J., Lark S., Williams M.A., Muller D., Harrex H. (2018). Sleep and adiposity in preadolescent children: The importance of social jetlag. Child. Obes..

[18] Quan S.F., Combs D., Parthasarathy S. (2018). Impact of sleep duration and weekend oversleep on body weight and blood pressure in adolescents. Southwest J. Pulm. Crit. Care.

[19] Seicean A., Redline S., Seicean S., Kirchner H.L., Gao Y., Sekine M., Zhu X., Storfer-Isser A. (2007). Association between short sleeping hours and overweight in adolescents: Results from a US Suburban High School survey. Sleep Breath.

[20] Hickie I., He J., Lamers F., Merikangas K., Paksarian D., Zhang J. (2017). Sleep Patterns and Mental Health Correlates in US Adolescents. J. Pediatr..

[21] Jankowski K.S. (2015). Is the shift in chronotype associated with an alteration in well-being?. Biol. Rhythm Res..

[22] Cho B., Lim K. (2003). Development and validation of emotional or behavioral problems scale. Korean J. Christ. Couns..

[23] Kim S.-H., Kim K.-Y. (1998). Development of behavior problem scale for children and adolescence. J. Korean Home Manag. Assoc..

[24] Kim K., Won H., Lee J., Kim K. (1978). Standardization study of symptom check list-90 in Korea I: Characteristics of normal responses. J. Korean Neuropsychiatr. Assoc..

[25] Korea Disease Control and Prevention Agency Commentary for 2017 Korean National Growth Charts. https://knhanes.kdca.go.kr/knhanes/sub08/sub08_02.do.

[26] Hox J. (2010). Multilevel Analysis: Techniques and Applications.

[27] Matos M., Gaspar T., Tomé G., Paiva T. (2016). Sleep variability and fatigue in adolescents: Associations with school-related features. Int. J. Psychol..

[28] Agostini A., Carskadon M.A., Dorrian J., Coussens S., Short M.A. (2017). An experimental study of adolescent sleep restriction during a simulated school week: Changes in phase, sleep staging, performance and sleepiness. J. Sleep Res..

[29] Lo J.C., Lee S.M., Teo L.M., Lim J., Gooley J.J., Chee M.W. (2017). Neurobehavioral impact of successive cycles of sleep restriction with and without naps in adolescents. Sleep.

[30] Richardson C., Oar E., Fardouly J., Magson N., Johnco C., Forbes M., Rapee R. (2019). The moderating role of sleep in the relationship between social isolation and internalising problems in early adolescence. Child Psychiatry Hum. Dev..

[31] Wheaton A.G., Chapman D.P., Croft J.B. (2016). School start times, sleep, behavioral, health, and academic outcomes: A review of the literature. J. Sch. Health.

[32] Roenneberg T., Kuehnle T., Juda M., Kantermann T., Allebrandt K., Gordijn M., Merrow M. (2007). Epidemiology of the human circadian clock. Sleep Med. Rev..

[33] Kurina L.M., Knutson K.L., Hawkley L.C., Cacioppo J.T., Lauderdale D.S., Ober C. (2011). Loneliness is associated with sleep fragmentation in a communal society. Sleep.

[34] Mateo M.J.C., Díaz-Morales J.F., Barreno C.E., Prieto P.D., Randler C. (2012). Morningness-eveningness and sleep habits among adolescents: Age and gender differences. Psicothema.

[35] Yong M., Fischer D., Germann C., Lang S., Vetter C., Oberlinner C. (2016). Are chronotype, social jetlag and sleep duration associated with health measured by Work Ability Index?. Chronobiol. Int..

[36] Baum K.T., Desai A., Field J., Miller L.E., Rausch J., Beebe D.W. (2014). Sleep restriction worsens mood and emotion regulation in adolescents. J. Child Psychol. Psychiatry.

[37] Dahl R.E., Gunnar M.R. (2009). Heightened stress responsiveness and emotional reactivity during pubertal maturation: Implications for psychopathology. Dev. Psychopathol..

[38] Foundation N.S. Sleep in America Poll: 2020 Sleepiness and Low Levels of Action. https://www.sleepfoundation.org/professionals/sleep-america-polls/2020-sleepiness-and-low-action.

[39] Noh J.-W., Kim J., Cheon J., Lee Y., Kwon Y.D. (2020). Relationships between Extra-School Tutoring Time, Somatic Symptoms, and Sleep Duration of Adolescent Students: A Panel Analysis Using Data from the Korean Children and Youth Panel Survey. Int. J. Environ. Res. Public Health.

[40] Torsheim T., Wold B. (2001). School-related stress, school support, and somatic complaints: A general population study. J. Adolesc. Res..

[41] Dong L., Martinez A.J., Buysse D.J., Harvey A.G. (2019). A composite measure of sleep health predicts concurrent mental and physical health outcomes in adolescents prone to eveningness. Sleep Health.

[42] Depner C.M., Melanson E.L., Eckel R.H., Snell-Bergeon J.K., Perreault L., Bergman B.C., Higgins J.A., Guerin M.K., Stothard E.R., Morton S.J. (2019). Ad libitum weekend recovery sleep fails to prevent metabolic dysregulation during a repeating pattern of insufficient sleep and weekend recovery sleep. Curr. Biol..

[43] Simpson N.S., Diolombi M., Scott-Sutherland J., Yang H., Bhatt V., Gautam S., Mullington J., Haack M. (2016). Repeating patterns of sleep restriction and recovery: Do we get used to it?. Brain Behav. Immun..

[44] Díaz-Morales J.F., Jankowski K.S., Vollmer C., Randler C. (2013). Morningness and life satisfaction: Further evidence from Spain. Chronobiol. Int..

[45] Gau S.-F., Soong W.-T. (2003). The transition of sleep-wake patterns in early adolescence. Sleep.

